# RETRACTION: Effect of Rapid Rehabilitation Care on Surgical Site Wound Infection and Pain in Patients With Intertrochanteric Femoral Fractures: A Meta‐Analysis

**DOI:** 10.1111/iwj.70285

**Published:** 2025-03-03

## Abstract

**RETRACTION:** LiJ.‐H. , LiP. , and LiY. , “Effect of Rapid Rehabilitation Care on Surgical Site Wound Infection and Pain in Patients with Intertrochanteric Femoral Fractures: A Meta‐Analysis,” International Wound Journal 21, no. 4 (2024): e14540, 10.1111/iwj.14540.38069603 PMC10961049

The above article, published online on 09 December 2023, in Wiley Online Library (http://onlinelibrary.wiley.com/), has been retracted by agreement between the journal Editor in Chief, Professor Keith Harding; and John Wiley & Sons Ltd. Following an investigation by the publisher, all parties have concluded that this article was accepted solely on the basis of a compromised peer review process. The editors have therefore decided to retract the article. The authors did not respond to our notice regarding the retraction.

**RETRACTION:** J.‐H. Li , P. Li , and Y. Li , “Effect of Rapid Rehabilitation Care on Surgical Site Wound Infection and Pain in Patients with Intertrochanteric Femoral Fractures: A Meta‐Analysis,” International Wound Journal 21, no. 4 (2024): e14540, 10.1111/iwj.14540.38069603 PMC10961049

The above article, published online on 09 December 2023, in Wiley Online Library (http://onlinelibrary.wiley.com/), has been retracted by agreement between the journal Editor in Chief, Professor Keith Harding; and John Wiley & Sons Ltd. Following an investigation by the publisher, all parties have concluded that this article was accepted solely on the basis of a compromised peer review process. The editors have therefore decided to retract the article. The authors did not respond to our notice regarding the retraction.

